# Supplementary material for: A theoretical analysis of complex armed conflicts
Source: PLoS One. 2022 Mar 4;17(3):e0264418. doi: 10.1371/journal.pone.0264418 (PMC8896697; doi:10.1371/journal.pone.0264418)
Supplement: S1 File — (DOCX) [file pone.0264418.s001.docx]

**S1 File**

**APPENDIX 1** – *Derivation of eq. (3)*

For simplicity, we consider the case $x_{l}=0$, i.e., a conflict between two groups.

Assuming that the time $T_{i}^{'}$ spent in one unit of time by each unit of *i* to discover $l_{ij}$ units of *j* is inversely proportional to the size $x_{j}$ of group *j*, we can write

$$T_{i}^{'}=\frac{k_{i}}{x_{j}}l_{ij}$$

Moreover, assuming that the time $T_{i}^{''}$ spent in services by each unit of group *i* in one unit of time is simply proportional to $l_{ij}$, we can write

$$T_{i}^{''}=s_{ij}l_{ij}$$

Since $T_{i}^{'}+T_{i}^{''}=1$ we obtain

$$l_{ij}={x_{j}}/{(k_{i}{+s}_{ij}x_{j})}$$

which is eq. (3) in the case $x_{l}=0$. The extension to the case $x_{l}\neq0$ is left to the reader.

**APPENDIX 2** – *Sizes and losses are equivalent*

In this appendix we show that in conflicts between two groups the pair $x=(x_{1},x_{2})$ is in a one-to-one relationship with the pair $L=(L_{21},L_{12})$.

For this, let us write

$$L\left( x \right)=\left| \begin{matrix} L_{21}(x_{1},x_{2}) \\ L_{12}(x_{1},x_{2}) \end{matrix} \right|=\left| \begin{matrix} {x_{1}x_{2}}/{(k_{2}+s_{21}x_{1})} \\ {x_{1}x_{2}}/{(k_{1}+s_{12}x_{2})} \end{matrix} \right|$$

and consider the Jacobian matrix $\left[ {\partial L}/{\partial x} \right]$ which turns out to be given by

$$\left[ \frac{\partial L}{\partial x} \right]=\left| \begin{matrix} {k_{2}x_{2}}/{{(k_{2}+s_{21}x_{1})}^{2}} & {x_{1}}/{(k_{2}+s_{21}x_{1})} \\ {x_{2}}/{(k_{1}+s_{12}x_{2})} & {k_{1}x_{1}}/{{(k_{1}+s_{12}x_{2})}^{2}} \end{matrix} \right|$$

Hence

$$det\left[ \frac{\partial L}{\partial x} \right]={{k_{1}k}_{2}{x_{1}x}_{2}}/{{(k_{2}+s_{21}x_{1})}^{2}{(k_{1}+s_{12}x_{2})}^{2}-{x_{1}x_{2}}/{\left( k_{2}+s_{21}x_{1} \right)\left( k_{1}+s_{12}x_{2} \right)=}}$$

$$=\left[ {{x_{1}x}_{2}}/{(k_{2}+s_{21}x_{1})}(k_{1}+s_{12}x_{2}) \right]\left[ {{k_{1}k}_{2}}/{\left( k_{2}+s_{21}x_{1} \right)\left( k_{1}+s_{12}x_{2} \right)-1} \right]$$

Since both squared brackets are nonzero for strictly positive values of $x_{1}$ and $x_{2}$, we can conclude that the matrix $\left[ {\partial L}/{\partial x} \right]$ is nonsingular so that $x$ and $L$ are in a one-to-one relationship.

All the numerical experiments we have performed in conflicts between three or more groups suggest that this result holds in general. However, a formal proof is not yet available.

**APPENDIX 3** – *Parameter values*

*Figures 3,4* $b_{2}=1, c_{1}=5, k_{1}=0.5, k_{2}=0.4, s_{21}=1, s_{12}=1, \rho_{1}=0.02$*,*$\rho_{2}=0.02, R_{1}^{\max}=4, R_{2}^{\max}=4$*.*

*Figure 5* $b_{1}=2, b_{2}=1, c_{1}=5, c_{2}=1, k_{1}=0.05, k_{2}=0.4, s_{21}=1, s_{12}=1, \rho_{1}=0.5, \rho_{2}=0.1, R_{2}^{\max}=4$

*Figures 6-11* $b_{1}=5.8, b_{2}=-0.4, c_{1}=0.9, c_{2}=0.02, k_{1}=3, k_{2}=0.25, s_{21}=0.07, s_{12}=0.01, \rho_{1}=0.01, \rho_{2}=0.3, R_{1}^{max}=5, R_{2}^{max}=10$

*Figure 12* $b_{1}=-0.4, b_{2}=-0.4, c_{1}=0.9, c_{2}=0.1, k_{1}=0.025, k_{2}=0.025$*,*

$$s_{21}=0.4, s_{12}=0.4, R_{1}^{max}=1, R_{2}^{max}=1$$

*Figures 14, 15* $b_{1}=1.5, b_{2}=-1.2, b_{3}=-1.2, c_{1}=2.7, c_{2}=2.7, c_{3}=0.9, k_{1}=0.3$*,* $k_{2}=0.007, k_{3}=0.005, s_{12}=0.13, s_{21}=0.04, s_{23}=0.13, s_{32}=0.13$*,*

$$\rho_{1}=0.05, \rho_{2}=3, \rho_{3}=3.2, R_{1}^{max}=15, R_{2}^{max}=3.6, R_{3}^{max}=3$$

*Figure 16* $b_{1}=1.5, b_{2}=-1.2, b_{3}=-1.2, c_{1}=2.7, c_{2}=2.7, c_{3}=0.9, k_{1}=0.3$*,* $k_{2}=0.007, k_{3}=0.005, s_{12}=0.13, s_{21}=0.04, s_{23}=0.13, s_{32}=0.13$*,*

$$\rho_{1}=0.5, \rho_{2}=3, \rho_{3}=3.2, R_{1}^{max}=15, R_{2}^{max}=3.6, R_{3}^{max}=3$$

*Figure 17(a)-(c)* $b_{1}=-0.5, b_{2}=6.5, b_{3}=-0.1, c_{1}=0.2, c_{2}=0.9, c_{3}=0.2, k_{1}=0.025, k_{2}=3.12, k_{3}=0.025, s_{12}=0.07, s_{21}=0.1, s_{23}=0.1, s_{32}=0.007$*,*

$$\rho_{1}=0.03, \rho_{2}=0.01, \rho_{3}=0.03, R_{1}^{max}=1.5, R_{2}^{max}=5, R_{3}^{max}=0.5$$

*Figure 17(d)-(f)* $b_{1}=-0.4, b_{2}=-0.4, b_{3}=0.5, c_{1}=0.9, c_{2}=0.2, c_{3}=0.9, k_{1}=0.025, k_{2}=0.025, k_{3}=0.025, s_{12}=0.4, s_{13}=0.12, s_{21}=0.4, s_{23}=0.03$*,*

$$s_{31}=0.4, s_{32}=0.15, \rho_{1}=3.37, \rho_{2}=3.5, \rho_{3}=0.7, R_{1}^{max}=1, R_{2}^{max}=1, R_{3}^{max}=5$$

*Figure 18* $b_{1}=8, b_{2}=8, b_{3}=-0.4, b_{4}=-0.4, b_{5}= -0.4, c_{1}=0.9, c_{2}=0.9$*,* $c_{3}=0.45, c_{4}=0.28, c_{5}=0.45, k_{1}=3, k_{2}=3, k_{3}=0.025, k_{4}=0.025, k_{5}=0.025, s_{13}=8, s_{14}=8, s_{24}=8, s_{25}=8, s_{31}=0.007, s_{41}=0.007, s_{42}=0.007, s_{52}=0.007, \rho_{1}=0.01, \rho_{2}=0.01, \rho_{3}=0.032, \rho_{4}=0.04$*,*

$$\rho_{5}=0.032, R_{1}^{max}=5, R_{2}^{max}=5, R_{3}^{max}=1, R_{4}^{max}=1, R_{5}^{max}=1$$

*Figures 19, 22* $b_{1}=6.87, b_{2}=-0.4, c_{1}=0.9, c_{2}=0.055, k_{1}=3, k_{2}=0.25, s_{21}=0.07, s_{12}=0.01, \rho_{1}=0.01, \rho_{2}=0.3, R_{1}^{max}=5, R_{2}^{max}=10$

*Figures 21, A5* $b_{1}=1, b_{2}=-0.2, c_{1}=0.1, c_{2}=0.05, k_{1}=32, s_{21}=0.05, s_{12}=0.4$*,*

$$\rho_{1}=0.1, \rho_{2}=0.3, R_{1}^{max}=1.25, R_{2}^{max}=0.25$$

*Figure 24* $b_{1}=5.8, b_{2}=-0.4, c_{1}=0.9, c_{2}=0.1, k_{1}=3, k_{2}=0.25, s_{21}=0.07$*,*

$$s_{12}=0.01, \rho_{1}=0.01, \rho_{2}=0.3, R_{1}^{max}=5, R_{2}^{max}=10$$

*Figure 25* $b_{1}=6.35, b_{2}=-0.4, b_{3}=5.5, b_{4}=-0.4, c_{1}=0.9, c_{2}=0.134, c_{3}=0.9, c_{4}=0.2, k_{1}=3, k_{2}=0.025, k_{3}=3, k_{4}=0.025, s_{12}=0.1, s_{21}=0.007, s_{34}=0.1, s_{43}=0.007, \rho_{1}=0.01, \rho_{2}=0.03, \rho_{3}=0.01, \rho_{4}=0.03$*,* $R_{1}^{max}=5, R_{2}^{max}=1, R_{3}^{max}=5, R_{4}^{max}=1$

**APPENDIX 4** – *Periodic stalemates do not exist in D – D conflicts*

Consider a degenerate *D* – *D* conflict with $b_{1}=b_{2}=0$. In such a conflict the origin is the unique non-negative equilibrium. Thus, positive cycles or homoclinic loops cannot exist in degenerate *D* – *D* conflicts because there are no strictly positive equilibria in the first quadrant (Poincaré rule). If $b_{1}$ and $b_{2}$ are slightly increased, the *D* – *D* conflict is not anymore degenerate and has only two nontrivial positive equilibria (${b_{1}}/{c_{1}}$ and ${b_{2}}/{c_{2}}$) on the axis, so that still there are no positive cycles or homoclinic loops. If $b_{1}$ and $b_{2}$ are further increased, strictly positive equilibria can appear through *TC* bifurcations of the two equilibria on the axis or through *SN* bifurcations of equilibria (see state portraits in Fig. 3). However, each time this happens, the new strictly positive equilibrium is connected through trajectories with at least two other equilibria (see state portraits in Fig. 3). Thus, a net of trajectories and equilibria connects the two positive semi-axis of the state portrait. This prevents that varying $b_{1}$ and $b_{2}$ a positive cycle or homoclinic loop suddenly appears in the state portrait through a tangent bifurcation of cycles or through a homoclinic bifurcation. In fact, any compact region delimited by a closed regular boundary (a candidate cycle) in the positive quadrant either intersects trajectories of the net or does not contain equilibria and in both cases this implies that the boundary of the compact region cannot be a cycle. But, in principle, positive cycles can appear through Hopf bifurcations. To prove that also this is not possible we now show that Hopf bifurcations cannot exist.

The model of a *D* – *D* conflict is a two-dimensional dynamical system

$$\dot{x}_{1}=F_{1}(x_{1},x_{2})$$

$$\dot{x}_{2}=F_{2}(x_{1},x_{2})$$

so that its Jacobian matrix *J* is given by

$$J=\left| \begin{matrix} \frac{\partial F_{1}}{\partial x_{1}} & \frac{\partial F_{1}}{\partial x_{2}} \\ \frac{\partial F_{2}}{\partial x_{1}} & \frac{\partial F_{2}}{\partial x_{2}} \end{matrix} \right|$$

The existence conditions of Hopf bifurcations are (see, e.g., [21, 22])

$$tr J=0\det J>0$$

at an equilibrium of the system. The first condition

$$tr J=\frac{\partial F_{1}}{\partial x_{1}}+\frac{\partial F_{2}}{\partial x_{2}}=0$$

implies that the two terms on the diagonal of the Jacobian matrix are of opposite sign, so that

$$\frac{\partial F_{1}}{\partial x_{1}} \frac{\partial F_{2}}{\partial x_{2}}\leq0$$

Thus, in view of this inequality the second condition

$$\det J=\frac{\partial F_{1}}{\partial x_{1}} \frac{\partial F_{2}}{\partial x_{2}}-\frac{\partial F_{1}}{\partial x_{2}} \frac{\partial F_{2}}{\partial x_{1}}>0$$

cannot be satisfied if the mixed derivatives ${\partial F_{1}}/{\partial x_{2}}$ and ${\partial F_{2}}/{\partial x_{1}}$ have the same sign.

But

$$F_{1}\left( x_{1},x_{2} \right)=b_{1}x_{1}-c_{1}x_{1}^{2}+{\rho_{1}R_{1}^{max}L_{21}}/{\left( \rho_{1}L_{21}+R_{1}^{max} \right)-L_{21}}$$

so that (after some algebra) it follows that

$$\frac{\partial F_{1}}{\partial x_{2}}=-\left[ \left( {\partial L_{21}}/{\partial x_{2}} \right)/{\left( \rho_{1}L_{21}+R_{1}^{max} \right)^{2}} \right]\left[ \left( R_{1}^{max} \right)^{2}\left( 1-\rho_{1} \right)+\rho_{1}^{2}L_{21}^{2}+2\rho_{1}L_{21}R_{1}^{max} \right]$$

Since $\left( {\partial L_{21}}/{\partial x_{2}} \right)$ is positive and $\rho_{1}<1$, the mixed derivative ${\partial F_{1}}/{\partial x_{2}}$ is negative. Obviously, the same is true for the other mixed derivative. Thus, the determinant of the Jacobian matrix cannot be positive and this allows us to conclude that cycles do not exist in *D* – *D* conflicts.

**APPENDIX 5** – *Seasonalities can be destabilizing*

The problem of chaotic stalemates in conflicts between two groups affected by environmental stress can be discussed by casting the problem in a general conceptual frame.

For this we assume that the dynamics of the environmental stress *w*(*t*) are described by a finite number, say *m*, of ODEs. Thus, the complete model of the conflict has the structure shown in Fig. 21 and is described by (*m*+2) ODEs

$\dot{w}\left( t \right)=g(w\left( t \right),p)$ (A5.1)

$\dot{x}\left( t \right)=f(x\left( t \right),w\left( t \right),q)$ (A5.2)

where *p* and *q* are constant parameters. The Jacobian matrix of the system

$$J=\left| \begin{matrix} \frac{\partial g}{\partial w} & 0 \\ \frac{\partial f}{\partial w} & \frac{\partial f}{\partial x} \end{matrix} \right|$$

has therefore a triangular structure.

The dynamics of the environmental stress are concisely captured by the so-called Lyapunov exponents [21, 52], which are *m* real numbers, that depend on the parameter *p* which characterizes the attractor (assumed to be unique) of the environmental submodel (A5.1). They describe the sensitivity of *w*(*t*) to the *m* components of the initial condition *w*(0) and can be estimated numerically by suitably averaging the *m* x *m* time-varying matrix [∂*g*/∂*w*] along a solution of (A5.1) (in the following, we use the algorithm based on QR-decomposition proposed by [75]).

Positive Lyapunov exponents reveal the divergence of nearby solutions, typical of chaotic regimes, while negative Lyapunov exponents reveal their convergence. The sign of the largest Lyapunov exponent (LLE) identifies the nature of the attractor: chaotic regimes have positive LLEs, periodic and quasi-periodic regimes have zero LLEs, and stationary regimes have negative LLEs. In the following, the LLE of the environmental submodel (A5.1) is indicated by $L_{g}$ and called environmental Lyapunov exponent.

The Lyapunov exponents of the conflict, i.e., the Lyapunov exponents of the complete model are therefore (*m* + 2) real numbers that can be extracted from the Jacobian matrix evaluated along a solution (*w*(*t*), *x*(*t*)) of model (A5.1, A5.2). In view of the triangular structure of the Jacobian matrix, *m* Lyapunov exponents of the complete model are those of the environmental submodel (A5.1), while the two remaining exponents are generated by the “military” submodel, that is, by the Jacobian [∂*f*/∂*x*]. These two Lyapunov exponents, generated by the intrinsic conflict dynamics and here called military, depend not only on *q* but also on the parameter *p* because the matrix [∂*f*/∂*x*] depends on the environmental stress *w*(*t*). Thus, the largest military Lyapunov exponent, indicated by $L_{f/g}$, is actually conditioned to the characteristics *p* of the environmental stress. That is why it is called conditional.

Let us now assume that the environment is chaotic ($L_{g}$ > 0). As for the largest military Lyapunov exponent, we consider the following two cases:

(a) $L_{f/g}$ > 0 (b) $L_{f/g}$ < 0,

that is, we intentionally rule out from our analysis the particular case $L_{f/g}$ = 0, which does not occur generically if the environment is chaotic (this is not so if the environment is periodic). In all cases, the conflict (i.e., the complete model) is chaotic because its largest Lyapunov exponent $L_{f, g}$ is positive, since

$$L_{f,g}=max\left\{ L_{g},L_{f/g} \right\}\geq L_{g}>0$$

In case (a), the military submodel reinforces environmental chaos, while in case (b), the military submodel does not contribute to the complex behavior of the conflict, which is exclusively due to the environment. For this reason we say that in case (a) there is military chaos, while in case (b) there is no military chaos because the sizes of the groups simply respond to the environmental stress. The sign of $L_{f/g}$ is therefore the only information needed for detecting whether chaos in a conflict is simply entrained by the environment or is, at least in part, generated by the recruitment policies and military characteristics of the groups.

We now study is some detail a conflict that can have military chaos for suitable parameter values. For this we consider a *D*–*A* conflict that we have already studied when the environmental stress is absent.

Our target is to point out that military chaos can emerge when the conflict is affected by seasonal environmental stresses. For this, we consider the case in which the environmental stress is periodic, as this assumption simplifies the analysis. In fact, if the environment varies periodically, i.e., if the attractor of the environmental submodel (A5.1) is a cycle, the largest environmental Lyapunov exponent $L_{g}$ is equal to zero, so that $L_{f,g} = L_{f/g}$. In other words, if the environment varies periodically, military chaos exists only if the conflict is chaotic.

We now proceed by focusing on a particular case (without claiming that it is particularly meaningful). For this, we assume that the killing time $k_{2}$ of the attack group varies sinusoidally over time, peaking once per year, i.e.,

$k_{2}(t)=\bar{k}_{2}(1+\varepsilon sin\frac{2\pi}{T}t)$ $0\leq\varepsilon\leq1$

where $\bar{k}_{2}$ and $\varepsilon$ represent the mean value and the relative variation of the killing time of the *A* group and *T* = 52 if the time unit is the week.

To see if military chaos exists, we simulate the model for various values of the pair (*ε*,$\bar{k}_{2}$). Each simulation must be very long because its initial part (the transient toward the attractor) must be disregarded if we wish to detect if the sizes (or the losses) become, in the long term, periodic, quasi-periodic, or chaotic.

The results of these simulations are long time series of the sizes. Figure A5.1 shows, for example, in its left panels, the evolution of the size $x_{1}(t)$ of the *D* group for the five different pairs (*ε*,$\bar{k}_{2}$) indicated in the caption. It is easy to recognize that the first three time series are periodic and that the last two are not, but it is difficult, if not impossible, to say from simple inspection, if they are quasi-periodic or chaotic. Extracting from each long time series the peaks $x_{1}^{h}$, *h* = 1, 2, 3, . . . and plotting them one versus the previous one, we obtain the so-called peak-to-peak plot (PPP), which is a visualization of the attractor on a Poincaré section [48]. Thus, if the PPP contains only one point (or a few points) the time series is periodic because a cycle intersects a Poincaré section at a single point (or at a few points). In contrast, if the points of the PPP are a regular curve (the section of a torus) the time series is quasi-periodic, while if the PPP is a fractal set the time series is chaotic. The PPPs of the five time series are shown in the right panels of Figure A5.1. The first three allow one to confirm that the corresponding time series are periodic with 2, 5, and 3 peaks per period (note that all periods are equal to 1 year except the second which is equal to 2 years), while the last two allow one to understand that the corresponding time series are quasi-periodic and chaotic.

To detect the nature of each time series one can complement the simulations with an algorithm for the evaluation of Lyapunov exponents. For example, the algorithm of [75] gives for the six cases of Figure A5.1 the following estimates of the largest military Lyapunov exponent

1. $L_{f/g}=-0.005$, (b) $L_{f/g}=-0.0007$, (c) $L_{f/g}=-0.005$,

(d) $L_{f/g}=0.000001$, (e) $L_{f/g}=0.018$

The signs of these five estimates and the fact that the fourth is practically zero reveal that the first three time series are periodic, the fourth is quasi-periodic, and the last is chaotic, as already established from the PPPs of Figure A5.1.

To discuss the existence of military chaos more deeply we must compute the largest military Lyapunov exponent more systematically for many parameter settings and extract general messages from the results. A simple way is to fix all parameters except two and evaluate all Lyapunov exponents of the model (and, hence, also $L_{f/g}$) in a large region of the two-dimensional space of the free parameters. Then, this operation can be repeated for other free parameters, or at least for those that are suspected, for some reason, of playing some role in promoting military chaos. For example, in the case of our conflict, we may be interested in gaining insights in the role played by variance and mean of the killing time of the *A* group. If so, we must compute the largest military Lyapunov exponent $L_{f/g}$ on a dense grid covering a large region of the space (*ε*,$\bar{k}_{2}$).


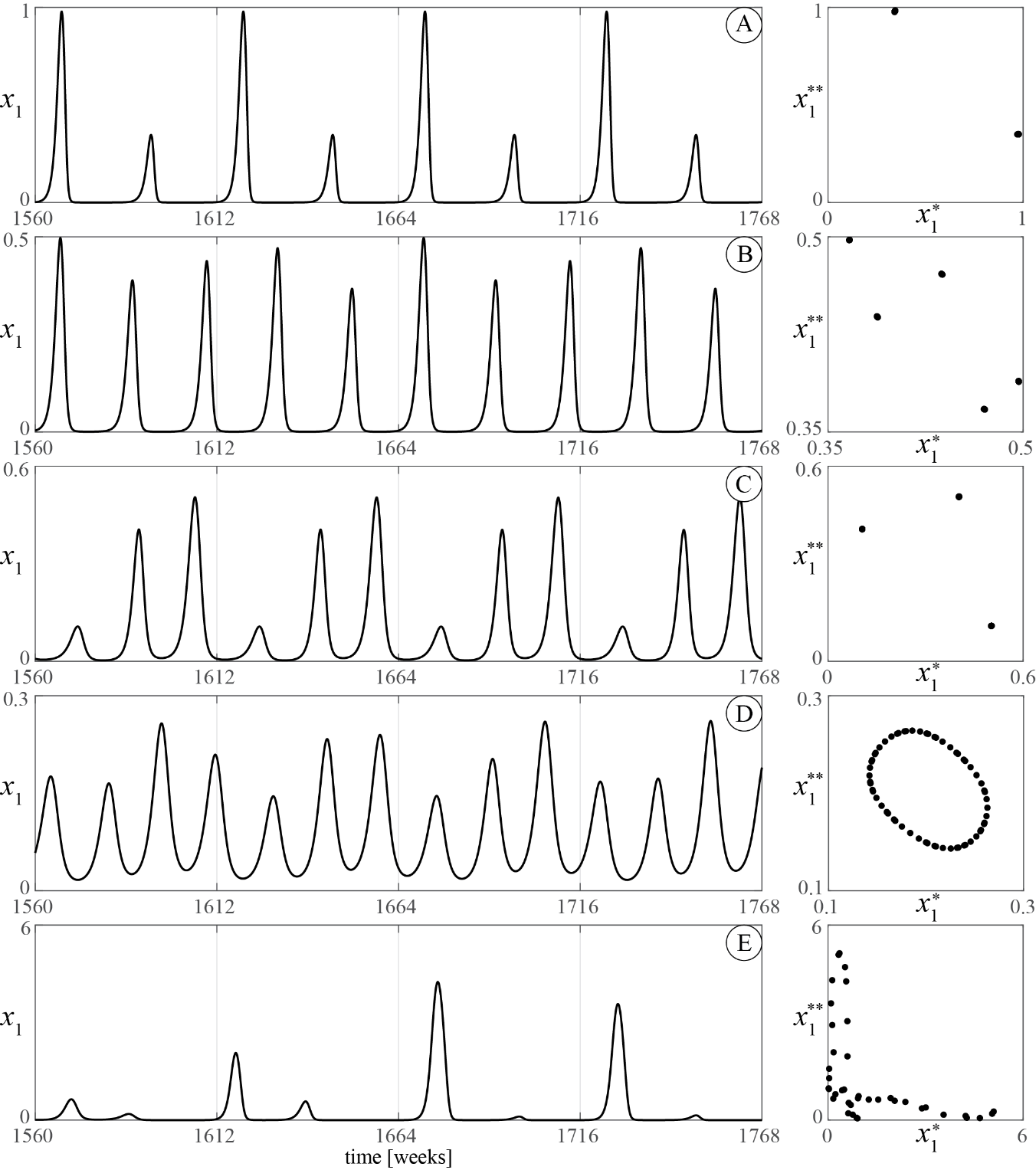


**Figure A5.1:** Four years long time series of the size $x_{1}$ of a *D* group obtained through simulation of the *D* – *A* model with parameter values given in Appendix 3 and $k_{2}$ varying sinusoidally as $k_{2}(t)=\bar{k}_{2}(1+\varepsilon sin\frac{2\pi}{T}t)$: (A) $\bar{k}_{2}=0.05$, $\varepsilon=0.3$; (B) $\bar{k}_{2}=0.06317$, $\varepsilon=0.1$; (C) $\bar{k}_{2}=0.085$, $\varepsilon=0.5$; (D) $\bar{k}_{2}=0.1$, $\varepsilon=0.2$; (E) $\bar{k}_{2}=0.08$, $\varepsilon=0.8$. On the right of each time series the corresponding PPP.

Before performing the computations it is worth asking what the theory of dynamical systems can tell us beforehand. If, for intuitive reasons, we imagine that the killing time is a stabilizing factor, we should expect that the model in the absence of seasons has a stable cycle for low values of $\bar{k}_{2}$ and a stable equilibrium for high values of $\bar{k}_{2}$. All this can be confirmed by a bifurcation analysis with respect to $\bar{k}_{2}$, with *ε* = 0. In particular, it can easily be established that the cycle exists for $\bar{k}_{2}<\bar{k}_{2}^{H}=0.11$ and that its period depends upon $\bar{k}_{2}$ as explicitly shown in Figure A5.2, where the points A, B, and C are those at which the period of the cycle is equal to 26, 20.8, and 17.3 weeks, that is, 1/2, 2/5, and 1/3 times the period of the environmental stress (equal to 52 weeks). This means that the environmental and the military clocks beat more and more synchronously when $\bar{k}_{2}$ is decreased.


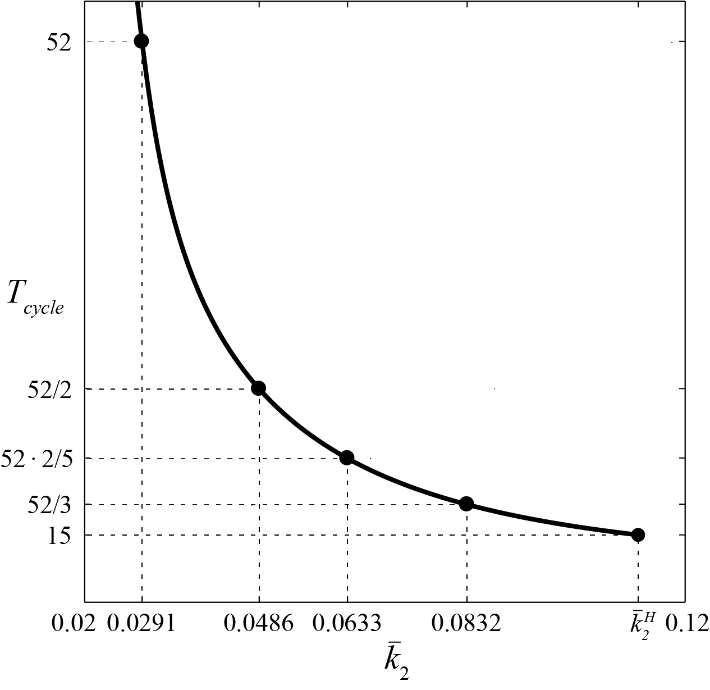


**Figure A5.2:** The period *T_cycle_* of the cycle in the absence of seasons decreases with $\bar{k}_{2}<\bar{k}_{2}^{H}=0.11$.

We can now introduce some small variability in the killing time of the *A* group, i.e., assume that *ε* is positive but small. If we are close to the upper part of the vertical axis in Figure A5.3, conflict can be approximated as linear (because *ε* is small) and affected by a sinusoidally varying stress. Thus, its response should simply be a sinusoidal response. In other words, for sufficiently low values of *ε* and $\bar{k}_{2}< \bar{k}_{2}^{H}$ we expect the conflict to have a periodic stalemate, as qualitatively indicated in Figure A5.3. Similarly, close to the vertical axis below the Hopf bifurcation point $\bar{k}_{2}^{H}$, the conflict is approximately a periodically varying linear system forced by a sinusoidal input and the expected behavior is quasi-periodic, as sketched in Figure A5.3. In conclusion, for small values of *ε* the behavior of the conflict is periodic if $\bar{k}_{2}$ is high and quasi-periodic if $\bar{k}_{2}$ is low. The boundary between these two regions, called *Neimark-Sacker* bifurcation curve, can, actually, be produced very precisely with continuation algorithms. But more can be said on the behavior of the conflict for *ε* small, as specified in Figure A5.3 where a few regions (called *Arnold’s tongues*) in which the regime is periodic are qualitatively sketched. When *ε* is very small, these regions are so tiny that they cannot be seen (the distance between their lower and upper boundaries is smaller than a pixel). Each Arnold’s tongue is rooted at a point $\bar{k}_{2}$ on the vertical axis, where for *ε* = 0 the ratio between the period of the cycle (indicated as $T_{cycle}$ in Figure A5.2) and the period of the environmental stress is a rational number, i.e.,


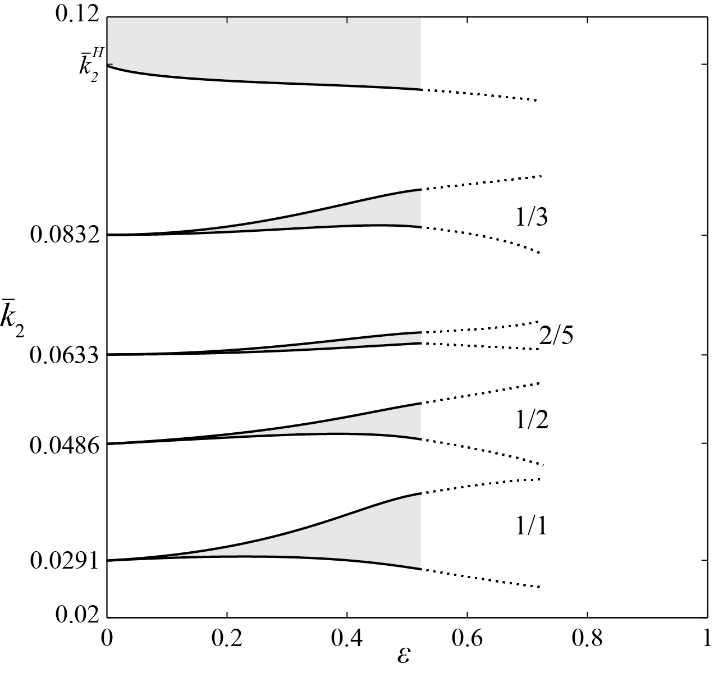


**Figure A5.3:** A sketch of the expected regimes for small values of *ε*: in the gray areas the expected stalemates are periodic, while they are quasi-periodic in the white areas below the Neimark-Sacker bifurcation curve *NS* rooted at point $\bar{k}_{2}=\bar{k}_{2}^{H}=0.11$ on the vertical axis.

${T_{cycle}}/{T_{env}}=p/q$ (A5.3)

where *p* and *q* are integer numbers. For example, Figure A5.2 shows the values of $\bar{k}_{2}$ for which *p*/*q* is equal to 1/2, 2/5, and 1/3.

Equation (A5.3) shows that in an interval of time equal to $pT_{env}=qT_{cycle}$ the environmental stress goes through *p* complete cycles while the conflict goes through *q* military cycles. In other words, recalling that $T_{env}=1$ year, the conflict has a *p* years long periodic behavior characterized by *q* (identical) peaks of the sizes. For example, if the value of $\bar{k}_{2}$ corresponds to point B in Figure A5.2, there are 5 military peaks every 2 years. If a small seasonality *ε* is introduced while $\bar{k}_{2}$ is not varied, one can expect that the *p* years long stalemate with *q* military peaks is not destroyed but that the *q* military peaks do not remain identical. Moreover, it can be shown that this rather special periodic behavior remains even if $\bar{k}_{2}$ is slightly perturbed (from the lower to the upper boundary of the tongue). This phenomenon, often referred to as *frequency locking*, is difficult to point out, not only empirically but also numerically, because the *p*/*q* tongues are very tiny, in particular if *p* and *q* are large.

Now that we have qualitatively seen what the theory suggests, we can look at the precise behavior of the conflict by numerically computing the largest military Lyapunov exponent $L_{f/g}$ on a dense grid in the space (*ε*,$\bar{k}_{2}$). The result, shown in Figure 21, is in very good agreement with all we said so far. First of all, a curve separates the upper green region (where the stalemate is periodic) from the large yellow region (where the stalemate is quasi-periodic). As expected, this curve is the Neimark-Sacker curve rooted at point $\bar{k}_{2}=\bar{k}_{2}^{H}$ on the vertical axis. Second, a great number of Arnold’s tongues, where the stalemates are periodic but locked on torus, are clearly visible. The largest of them are rooted at the points of the vertical axis corresponding to points A, B, and C in Figure A5.2 where *p*/*q* is, respectively, equal to 1/2, 2/5, and 1/3. Consistently, in the highest tongue the stalemates are one year long and have two peaks per year, as clearly indicated in Figure A5.1*a* corresponding to point A in Figure 21. Similarly, in the other two tongues there are *q* (i.e., 5 and 3) peaks per cycle, as shown in Figure A5.1*b* and *c*, corresponding to the points B and C in Figure 21.

But Figure 21 also points out red regions where the regime is chaotic (as seen in Figure A5.1*e* corresponding to point E in Figure 21). The fractal nature of these regions is due to phenomena that occur with strong seasonalities (*ε* large), and this is why it cannot be predicted from the theory concerning low seasonalities (*ε* small). Some interesting features of these chaotic regions could be discovered through standard bifurcation analysis, but we do not expand such an issue here, because this would bring us too deeply into the mathematical sphere. Instead, we note from Figure 21 that the red chaotic regions are regularly distributed in the space (*ε*,$\bar{k}_{2}$) and that the degree of seasonality ε required to generate chaos is lower for lower values of $\bar{k}_{2}$. But recalling Figure A5.2, we can also conclude that less seasonality *ε* is needed to generate chaos if the military and environmental clocks beat at comparable frequencies. This message is perhaps the most valuable conclusion emerging from this relatively complex discussion.
