# Supplementary material for: A theoretical analysis of complex armed conflicts
Source: PLoS One. 2022 Mar 4;17(3):e0264418. doi: 10.1371/journal.pone.0264418 (PMC8896697; doi:10.1371/journal.pone.0264418)
Supplement: Attachment — Submitted filename: response_reviewers.pdf [file pone.0264418.s003.pdf]

Reviewer #1: Dear Editor,

I have read the revised manuscript submitted by Rinaldi et al. I appreciate the work the authors have put into revising the manuscript, and I find it much improved.

One of my main concerns previously was that the connection between the mathematical results and the historical examples were too tenuous for the strength of the claims made in the paper. I find that the new presentation is much better and highlights the big picture without overly specific claims.

Given that neither the other reviewer nor I are historical experts, however, it seems important to verify the historical descriptions with another expert.

Another major concern I had was with the dearth of citations of the extensive literature on conflict modeling. My point was in reference to a balance must be struck with citing widely, relevancy and the length of the manuscript. While I still feel that some additional citations are missing, I trust that this is a result of careful and purposeful selection by the authors.

Finally, I found the grammar and language of the revised manuscript much improved. Thank you for proofreading this with an English speaker.

We are happy that the Reviewer appreciated the work we did for the revision. Effectively, balance the huge literature on conflict and the length limits of the manuscript was harsh. If the Reviewer has in mind some particular relevant citation we are missing, please tell it to us, so that we can try to add it (or explain why we had not added it).

There remain a couple sections where the grammar is unusually strange and many typos remain—namely pages 25 and 33-34.

We have rephrased page 25 and the last pages of the manuscript.

A few remaining questions and comments

Is there a particular reason for choosing the parameters b1 and c2 for Figure 3 instead, for example, of the ratios of these parameters b1/b2 and c1/c2 for the two models?

Unfortunately, this is not possible, because the ratios between two parameters do not uniquely identify the system dynamics. For example, let c1=5 and c2=2; if b1=b2=1 (b1/b2=1) the system's dynamics is described by the portrait 5, while if b1=b2=2 (b1/b2=1) the system's dynamics is described by the portrait 1.

In lines 29 and 41 “i.e.” is italicized and then not. I believe it does not need to be italicized, but this should at least be consistent.

Following the Reviewer suggestion, we have removed all the italic forms for ‘i.e.’ in the manuscript.

There are some sentences with extra spaces like in line 201 amongst other places. This should be an easy find and replace correction.

Done.

As the other reviewer mentioned, it is important to justify why the size of the army is the important variable.

The size of the group, as stated in the paper, is a suitable variable capturing the group’s war ability. For this reason, we assume that we can use this variable to mathematically describe the conflict dynamics.

Line 73 should be “to the Appendix”

Line 371 do the authors mean “size” and not “dimension”?

Figure 19 typo “raising”

Line 569 “etc.” instead of “…”

Line 569 “the” unnecessary before “experience”

Line 572 no “their” before “damages”

Punctuation issues in lines 573-576

Lines 618-619 Is there more explanation of this “interesting result”?

Line 643 and elsewhere, conflicts are described as “wild”. This sounds rather colloquial to this native English speaker. I would suggest a different word.

Line 750, “terroristic attack” -> “terrorist attack”

Done, thank you.

Reviewer #2: I see that almost all of my comments addressed satisfactorily, I have two issues remaining:

(a) the text relating to operation Barba Rossa, see below

(b) military LE; I have a suggestion below, and leave the decision to the editor.

I read the version highlighting the changes and found a few typos still, the line numbers refer to that version.

p5 l145; bureaucratic--> bureaucracy

p6 l155 Richardson's models

156-157; models by Lanchester (ref) and Deitchman (reads nicer)

p7 l182 less-> lower

l189 an epidemic (singular), not epidemics (plural)

l218 qualitative --> qualitatively

l261 forces about --> forces were about

310 Westmorelands' (it is *his* call, yes, I insist) *(Gotta! Even you misspelled his last name again! Thank you)*

l367 show --> shows

l381 large-N scholarship? I have no clue what you mean. Perhaps you mean large-N studies?

438 objective --> objectives

443-446; Seems an unfinished sentence to me.

502 this rules --> these rules

607 , ... reads as unfinished

808 have --> has

l1215; IBC; with online sources you must state what date you retrieved it.

We thank the Reviewer for his precise and punctual comments.

---

l265-6; use of tanks. When you continue with "such innovations" the flow of your story is interrupted, and hard to read. The tanks-part is isolated now. I cannot decide whether you mean both tanks and Stormtroopers or just the latter.

We thank the Reviwer for his comment. Effectively, we were trying to say to many things, and, at the end, we make confusion in the reader. Stromtroopers were the innovation that break stalemate for German, while innovation related to tanks (principally done by British) contribute to break another stalemate. In the revised version of the text, we now only talk about the impact of infantry tactics.

---

l341-6 May I suggest to rewrite this? For instance, the state of the Russian army initially was poor even if they had large numbers, and they had chosen a wrong tactic. The mass killing of officers earlier made warfare coordination for the Russians initially hard too. Reading on Barba Rossa, the brutalness came later, and the mass mobilization came later too when German troops had no ammo nor gas left and supplies were hard to get. You may even wonder why the Germans got that far at all. It appears to me that reality here is so complex that your 5-line description is not adequate.

We did rewrite and clarify making other factors contributing to early German success explicit (although in respect of word constraints). Yet, we maintained a key element of the previous version. While we don't deny that other factors contributed to German advance, and it was during the German retreat that most violence effectively occurred, "brutal" management of conquered territories clearly allowed Germans to speed up operations. This did not just occur while retreating (though this was more widespread and visible) but also while advancing. The September-October 1941 Babi Yar massacre – for instance – has been described as part of a broader strategy adopted by German forces to "eliminate pockets of resistance" (the point is made for instance by Shepherd, Ben, War in the Wild East: The German Army and Soviet Partisans, cited in the text but also by Wette, Wolfram, Wehrmacht. History, Myth, Reality, Harvard University Press, 2006 and present in the memoirs of some German officers, Bidermann, Gottlob Herbert. 2000. "In Deadly Combat: A German Soldier's Memoir of the Eastern Front, Trans." Derek Zumbro. Lawrence: Univ. Press of Kansas). Statements on Soviet resources mobilized comes from data available in Harrison, M. (1988). Resource mobilization for World War II: the USA, UK, USSR, and Germany, 1938-1945. Economic History Review, 171-192 (reference added).

In the appendix: I still think military LE is a wrong name, and should be avoided. Rather you would like to say this is the intrinsic LE opposed to the environmental LE as it is derived from the "conflict dynamics".

We do like the term 'military LE', that is in accordance also with the terms 'biological Lyapunov Exponent' (Colombo et al. 2008) and the terms 'romantic Lyapunov Exponent' (Rinaldi et al. 2015).
