# Supplementary material for: A theoretical analysis of complex armed conflicts
Source: PLoS One. 2022 Mar 4;17(3):e0264418. doi: 10.1371/journal.pone.0264418 (PMC8896697; doi:10.1371/journal.pone.0264418)
Supplement: Attachment — Submitted filename: response_reviewers.pdf [file pone.0264418.s004.pdf]

Reviewer #1; Dear Editor,

I think the manuscript has come a long way, and it is almost ready. I only have a few grammatical comments.

Line numbers refer to the manuscript with the deltas. Brackets indicate my edit.

Lines 151-153: Is there a parenthesis missing? "is unbounded" should be "[are] unbounded"

Line 360: "Many studies confirm[] that"

Figure 21 caption: Is not a more standard way to write "Points A, ..., E" instead "Points A-E"? There is also a period missing at the end.

Line 736: "concerns the idea [of trying] to interrupt"

Line 770: "focus[es] on" since the subject is "The most interesting study"

Sincerely,

Reviewer 1

Reviewer #2: In general, for the dynamical systems part I am satisfied. As for the interpretation, it is at least acceptable from my side. I must note however that the historical interpretations are not something I can really judge.

I suggest the peer reviews should be published such that it is clear that this was not checked by a historian.

I still think intrinsic and extrinsic Lyapunov Exponents is better terminology. Referring to a different paper that something similar was used is not a good argument. Again, I leave this to the editor.

I went through the text and especially the changes. I found one minor issue:

Line 235; looking at Figure 3 panel 2 I observe two saddles, one on the vertical axis, and a "positive" one within the first quadrant. So perhaps be even more careful indicating the "positive" one? Also, the trajectories forming the stable manifold will approach the saddle but never reach it. So I suggest to write "that approach the saddle".

All done. Just thank you!
